# Pilot Study of the Adaptation of an Alcohol, Tobacco, and Illicit Drug Use Intervention for Vulnerable Urban Young Adults

**DOI:** 10.3389/fpubh.2020.00314

**Published:** 2020-07-17

**Authors:** Tekeda F. Ferguson, Alaina Beauchamp, Erika M. Rosen, A. Nicole Ray, Katherine P. Theall, Nicholas W. Gilpin, Patricia E. Molina, Scott Edwards

**Affiliations:** ^1^Louisiana State University Health Sciences Center, Comprehensive Alcohol-HIV/AIDS Research Center, New Orleans, LA, United States; ^2^Department Epidemiology, Louisiana State University Health Sciences Center, School of Public Health, New Orleans, LA, United States; ^3^Neuroscience Program, Tulane Brain Institute, Tulane University, New Orleans, LA, United States; ^4^Department of Global Community Health and Behavioral Sciences, School of Public Health and Tropical Medicine, Tulane University, New Orleans, LA, United States; ^5^Department of Physiology, Louisiana State University Health Sciences Center, School of Medicine, New Orleans, LA, United States

**Keywords:** alcohol intervention, youth intervention, drug intervention, urban youth, drugs and tobacco in youth

## Abstract

**Objectives:** There is limited information about the applicability and effectiveness of tobacco and illicit drug use interventions in urban and racial/ethnic minority youth, a population with great need for prevention of alcohol and drug use. We pilot-tested the feasibility of a behavioral intervention to reduce alcohol, tobacco, and illicit drug use among urban young adults in New Orleans, Louisiana.

**Study Design:** The 12-week intervention pilot project was developed to be implemented at a community-based social service organization that provides educational, juvenile justice-related case management, and mentoring services to youth with substance use and incarceration histories.

**Methods:** One-hour intervention sessions included interactive discussions and lesson reviews guided by a health educator and peer facilitators. Recruitment was done by case managers. Thirty African American young adults aged 16–21 years participated between January 2016 and July 2017.

**Results:** We were able to adapt the 14-session intervention to a 12-session, weekly curriculum that was well-received by the target population. Average rating for each session was 9.5 ± 0.3 (scale 0–10). Youth were willing to engage in the program, but retention was low. Rates of alcohol and drug use were significantly higher within our pilot population than national estimates. We found no significant decreases in self-reported alcohol, tobacco, or illicit drug use after participation in the intervention.

**Conclusion:** Results emphasize the need to devote additional educational resources to intervention and retention factors for vulnerable youth. Individuals often experiment with drugs during adolescence; thus, this period represents a prime opportunity for education and intervention.

## Introduction

Substance use disorder (1) is one of the leading behavioral health issues among youth and young adults (2). Individuals often experiment with drugs during adolescence; thus, this period represents a prime opportunity for education and intervention. It has been shown that youth and young adults who abuse drugs are at a greater risk of developing patterns of harmful substance use and use disorders in adulthood (3). Therefore, the need for effective alcohol and drug prevention and intervention programs among youth and young adults remains a necessity in the US (4).

Disproportionately higher rates of alcohol and polysubstance-abuse-related harms are borne by out-of-school youth (5). Environmental, social, and psychological stressors (i.e., adolescent exposure to community violence) are excessive in urban areas and increase the risk of alcohol and drug abuse (6, 7). In Louisiana, 22% of the high school student population (grades 9–12) have reported trying their first drink of alcohol before the age of 13, compared to 16% across the United States. Among the same age group in Louisiana, 37% have used marijuana (compared to 36% in the US) and 10% have used cocaine (compared to 5% of the US) (8).

Cultivating positive motivation, cognitive skills, and social skills through mastery-focused interventions has conferred some degree of protection against substance use disorder among youth (9). Juvenile justice-involved youth and reentry populations are at increased risk of alcohol and substance abuse while also experiencing barriers to job and educational attainment (10). The fluid nature of reentry youth attendance in court-mandated programs presents unique challenges to conventional intervention delivery. Thus, the need for substance abuse education among this population warrants the continued development of effective programming (11).

The majority of evidence-based drug prevention interventions have been validated in largely suburban adolescent populations (6, 7), providing limited information about intervention applicability and effectiveness in urban and racial/ethnic minority youth. An analysis of the Blueprints for Healthy Youth Development (Blueprint) registry returned 83 evidence-based youth development and prevention programs that have been reviewed and validated (12). Thirty of the 83 programs were used to target problematic alcohol, tobacco, and illicit drug use. Only five programs in the Blueprint registry have been validated in a primarily African American (AA) population, and of these five programs only two were used to address alcohol, tobacco, or illicit drug use (13–15). The Strong African American Families (SAAF) program was an interactive group intervention performed as a family-centered program, which was not an ideal prevention strategy to use in our target population since our target was a non-family program for inner city youth and young adults in New Orleans, Louisiana (16).

The goals of our Comprehensive Alcohol-HIV/AIDS Research Center (CARC) Project Toward No Drug Abuse (TND) pilot project were to investigate the feasibility of implementing the TND Program for out-of-school, inner city youth and young adults and to evaluate the effect of the curriculum on drug use among participants in a novel community setting. Outcomes of interest included evaluating whether all components of the TND curriculum modules could be adapted for use in the target population and if participation in the CARC TND pilot program had any effect on self-reported substance use (e.g., alcohol, tobacco, marijuana). We hypothesized that participation in the program would decrease self-reported admission of illicit drug and alcohol use among enrolled youth compared to baseline reported levels.

## Methods

The Louisiana State University Health Sciences Center – New Orleans (LSUHSC-NO) Comprehensive Alcohol-HIV/AIDS Research Center (CARC) chose to adapt the Project Toward No Drug Abuse (TND) for use in an urban non-school-based program setting for young adults because it incorporates harm reduction principles and is a drug education curriculum that has shown statistically significant reductions in illicit drug and alcohol use in multiple randomized controlled trials (17). TND is based on the Motivation-Skills-Decision Making model, including elements of social learning theory to deliver knowledge about the transition from recreational substance use to addiction, and fosters practical skills associated with resiliency from addiction through group activities and guided discussion (18). The TND curriculum was developed at the University of Southern California (USC) and has been widely validated among alternative schools and traditional high school students in terms of reduction of alcohol and illicit drug use (19, 20).

The CARC TND pilot project was implemented by the LSUHSC-NO CARC and data were collected at the Youth Empowerment Project (YEP) in New Orleans, Louisiana from 2016 to 2017. The study protocol was reviewed and approved by the Institutional Review Board (IRB) of LSUHSC-NO and informed consent was obtained from all study participants.

### Study Population

Participants were recruited from a baseline population of ~120–130 out-of-school youth and young adults in New Orleans, Louisiana receiving YEP services at the time of the study. YEP is a community-based social service organization that provides case management services to underserved, out-of-school youth in the New Orleans metropolitan area through a variety of programs aimed at education (i.e., General Educational Development (GED) and job training) and job attainment. According to survey results from YEP in 2015, 56% of New Orleans youth report having ever used alcohol and/or drugs, and 47% reported that they felt the need to cut down on their use. We chose to use the YEP program for our CARC TND study population because the implementation of a tailored, evidence-based substance use prevention program that incorporates skills-training components in addition to regular programming was consistent with the New Orleans YEP mission and vision to “engage underserved young people through community-based education, mentoring and employment readiness programs to help them develop skills and strengthen ties to family and community” (21).

### Intervention

In this study, we adapted the TND schedule to complement a young adult learning program schedule at YEP (“The Village“) using health educators certified by TND developers, and incorporating peer mentors who were graduates of YEP programs. We designed the study curriculum to take place over a 12-week period and consist of 11 educational sessions that occurred every week during regularly scheduled program sessions, covering behavioral and cognitive antecedents of drug abuse. The 1-h sessions included interactive discussions and lesson reviews guided by a health educator and peer facilitators on presentation topics. Some sessions included myths and denials related to substance abuse, symptoms of chemical dependency, and emphasis on how substance abuse affects health (Table 1). As we adapted the TND curriculum for use, we collected qualitative feedback from focus groups conducted by the pilot study staff with participants and staff. External TND trainers and program evaluators reviewed the pre-planned CARC TND intervention and conducted an on-site group training prior to the start of our project and as well as reviewed all modifications/adaptations to the TND curriculum to ensure integrity and maximize fidelity.

**Table 1:** Weekly session topics of the Comprehensive Alcohol-HIV/AIDS Research Center (CARC), Toward No Drug Abuse (TND) 12-week pilot program and mean session evaluation score from participants.

**Week and topic**	**Session activities**	**Mean evaluation score**	**Participant compensation**
Week 1: Introduction and Pretest	Introduce project TND, explain study, consent, and consenting individuals complete the pre-intervention survey.		Tier 1: $10
Week 2: Active listening and stereotyping	Identify effective/ineffective communication strategies. Understand what stereotyping is and the implications of group-labeling. Emphasis will be placed on understanding the concept of “self-fulfilling prophecy.”	9.71	Tier 2: $12
Week 3: Myths and denials	Understanding how beliefs are used to justify actions and recognizing the “myths” of drug abuse.	9.89	
Week 4: Chemical dependency	Course of negative consequences associated with chemical dependency; information tailored to include local drug use trends and data.	9.64	Tier 3: $14
Week 5: Talk show (psychodrama)	Role-play as television talk show guests who are affected by drug abuse; explore the impact of drug abuse on family and friends as well as the substance abusing individual.	9.00	
Week 6: Marijuana panel and tobacco basketball	Familiarize youth with basic information about tobacco use and trends (game format).	9.20	Tier 4: $16
Week 7: Stress, health, and goals	Identify and define proactive and negative coping strategies and discuss how stress and drug use impacts health.	9.75	
Week 8: Self-Control	Learn about behavior-matching in the context of self-control; identify passive, aggressive, and assertive communication skills. Develop assertive communication skills.	9.20	Tier 5: $18
Week 9: Thought and behavior loops	Learn about thought process & behavior loops and how to recognize these patterns; e.g., violent behavior is a consequence of negative thoughts as a response to frustration/anger.	9.40	
Week 10: Perspectives	Identify cognitive dissonance and discuss strategies to align attitudes/behaviors.	9.80	Tier 6: $20
Week 11: Decision-making and commitment	Think through the options with respect to using drugs & violence, and outline life goals. Final activity will include signing a personal commitment statement as defined by participants for themselves.	9.40	
Week 12: Post-test and wrap-up	Complete the post-intervention survey. Participants received a certificate of completion recognizing their participation and mastery of skills and information presented in the intervention.		$20

Recruitment was carried out by YEP case managers, and three 12-week program sessions were delivered to sequential cohorts, taking place between January 2016 and July 2017. Participants were compensated for their time and participation in the study. Table 1 lists the tiers and amounts of compensation that were available and provided to participants, starting at $10. Youth who completed the pre-test and attended the first curriculum session were compensated $10. Participants must have attended two sessions (including pre-test) to qualify for tier 2 compensation of $12; until this requirement was met, compensation remained at $10. To progress in the $2 increment of compensation tiers, participants had to have increased attendance by two sessions. All youth were compensated $20 for completing the post-test. While no participants were excluded from receiving the intervention, inclusion criteria specified for data analysis was enrollment in YEP client services and completion of informed consent (including parental consent and assent of minors, if applicable).

Primary data collection was done using pre- and post-intervention test/survey questions from the National Institute of Drug Abuse-Monitoring the Future (NIDA-MTF) survey, which were collected during the first and last sessions, respectively. Session attendance was recorded for all participants and pre- and post-intervention survey data were collected via paper surveys. Surveys were delivered on-site at the YEP Adult Learning Center, entered electronically by YEP staff, and stripped of identifying information using Efforts To Outcomes (ETO) case management software. Each data entry record was checked and validated against the paper survey forms to correct any data entry errors. For each session, data were collected to assess fidelity and adaptation of the TND program, including participants' evaluations of each session and an evaluation of the facilitator's adherence to TND study protocol and quality of delivery were assessed by a staff member. The study staff members completing these evaluations were post-doctoral and master's level trained researchers, and on the days they were evaluating the session as an auditor, they only acted in that role and were not part of the intervention delivery. Items assessed included materials covered according to protocol, re-direction and prompts, and competence.

### Data Analysis

Descriptive analysis (e.g., mean, standard deviation) was carried out to assess the mean number of sessions attended by participants. Participant and facilitator evaluations of the TND curriculum were also assessed to determine sessions that were well-received in this population or sessions that need to be further refined for future TND implementations in demographically similar populations. Descriptive statistics were then used to characterize and summarize the survey data, and self-reported lifetime substance use was assessed and quantified for all TND participants. To evaluate whether there were changes in pre- and post-intervention substance use, we measured past 30-day use of alcohol, cigarettes, and marijuana in participants who completed both the pre- and post-intervention surveys.

In addition, we looked at whether self-reported substance use among the study population was similar to national estimates. As previously stated, several used in the pre- and post-intervention survey were from the NIDA-MTF survey; therefore, a direct comparison could be estimated. As our pilot study surveyed primarily African American youth, we compared the 2016 NIDA-MTF survey data for African Americans and African Americans in the Southern Metropolitan Statistical Areas (MSAs) to pre-survey responses from our CARC TND participants. Due to the age range of those who participated in this pilot study, we chose to look at NIDA-MTF data for 12th graders only instead of middle school data. Chi-square tests were used to determine statistically significant differences between substance use in national populations and the CARC TND pilot participants. All analyses were completed using SAS software version 9.4 and SAS survey analysis PROCs for analysis of national and regional weighted datasets.

## Results

Participants were receptive and found satisfaction in participation in the CARC TND intervention. A summary of each of the 12 sessions is listed in Table 1, along with the mean evaluation score from the young adult participants for each session with new content. The average rating across intervention sessions was 9.5 ± 0.3 standard deviation on a scale of 1–10. Session 3 (*Myths and Denials*) was the highest-rated session. The mean score was 9.89. Session 5 (*Talk Show*) had the lowest participant evaluation score (7.85 ± 2.61). This was due in part to low attendence rates, which were not ideal for panel-style discussions that required greater numbers of attendees. We found that during sessions with lower participation, pilot participants were less likely to engage in open discussion; the peer-counselors had to poll participants. All 12 sessions of the TND program were offered to all participants; however, the mean number of sessions attended was 5.6 ± 3.1. Fewer than half (43%) of enrolled youth participants completed 50% or more of the session, and 23.3% completed 75% or more of the intervention sessions, and this is a major limitation of this pilot study.

The CARC TND pilot study had a total sample size of 30 youth and young adults aged 16–21 years (mean age = 18.3 ± 1.3; Table 2). The pilot study population consisted of 36.7% under the age of 18 and 63% ≥18, with 63% males and 93% African American young adults. The percentage under and over 18 in our pilot sample was comparable to the NIDA-MTF African American sample from both the southern metropolitan statistical area (MSA) (*n* = 842) and the national NIDA-MTF (*n* = 1,699). The bottom of Table 1 provides the percentage of participants who reported ever using alcohol, cigarettes, vapor products, or marijuana in their lifetime among the CARC TND pilot participant compared to the national survey. Among TND participants, 73.3% indicated having ever used alcohol, 66.7% reported having ever used cigarettes, 43.3% had ever used vapor products, and 83.3% had ever used marijuana. Our pilot participants report higher use compared to the national estimates. Among African Americans in the Southern MSA region and national sample, alcohol and marijuana use were not different, with ~40% reporting having ever used these two substances. In the Southern MSA sample, 13.2% had used cigarettes and 0.06% had used vapor products. In the National sample of African Americans, 17.0% had used cigarettes and 0.05% had used vapor products.

**Table 2:** Demographics and lifetime self-report of ever-use of alcohol, tobacco, and marijuana among Project Toward No Drug Abuse (TND) pilot study participants, 2016 National Institute of Drug Abuse-Monitoring the Future (NIDA-MTF) African American (AA) participants from Southern Metropolitan Statistical Areas (MSAs), and all AA NIDA-MTF participants.

	**Project TND *N* = 30**	**AA NIDA-MTF southern MSA participants** *N* = 842^*^	**All AA NIDA-MTF** ***N*** **=** 1,699^*^	
Age (mean s.d.)	18.3 (1.3)	–	–	
Age group	% (n)	% (n)	% (n)	
Under 18	36.7 (11)	38.1 (321)	38.2 (648)	
≥ 18	63.3 (19)	61.6 (519)	61.6 (1,047)	
Missing	–	0.3 (2)	0.2 (4)	
**Sex**				
Male	63.3 (19)	42.4 (357)	44.8 (761)	
Female	36.7 (11)	51.5 (434)	48.4 (822)	
Missing	–	6.1 (51)	6.8 (116)	
**Race**				
Black	93.3 (28)	100.0 (842)	100.0 (1,699)	
White	3.3 (1)	–	–	
Other	3.3 (1)	–	–	
**Lifetime Drug Use**		**% (n)**		
Alcohol	73.3 (22)	39.3 (331)	40.6 (689)	^**^
Cigarettes	66.7 (20)	13.2 (111)	17.0 (289)	^**^
Vapor product	43.3 (13)	0.06 (47)	0.05 (84)	^**^
Marijuana	83.3 (25)	39.4 (332)	40.3 (684)	^**^

* weighted frequencies;

** *indicated p < 0.0001*.

When looking at self-reported lifetime use among the pilot participants, 26.7% had never used alcohol, while 36.7% had used it 1–2 times, and 13.3% had used alcohol in the highest category of 40 or more times (Table 3). Individuals aged 18 and older constituted a higher percentage who had ever used alcohol, but it was not statistically significant. Female participants had a statistically significant higher reported percentage of alcohol use than males. Estimates of ever-use are reported in Supplemental Table 1. Table 3 further shows self-reported lifetime cigarette, cigar, marijuana, and non-prescribed drug use overall, as well as by age and sex. Thirty percent (30.0%) of the pilot participants reported using cigarettes 40 or more times, while 33.3% had never used cigarettes. Individuals 18 years and older exhibited more cigarette and cigar/cigarillo use than those under 18, and males reported more use than females (neither trends were statistically significant). Cigar or cigarillo use was low with 66.7% of participants reporting never using these products, while 56.7% reported never using vaping products, and 76.7% reported never using non-prescribed prescription drugs in their lifetime. Marijuana was the most commonly self-reported substance used at baseline, with only 16.7% reporting never having used marijuana, while 53.3% reported using marijuana 40 or more times. There was a significant increase in self-reported marijuana use by individuals 18 and older (*p* = 0.023). There was no reported use of methamphetamines, heroin, hallucinogenic drugs, or steroids among CARC TND pilot study participants.

**Table 3:** Baseline number of times in the young adult's lifetime that substances were used by age group and sex among the Comprehensive Alcohol-HIV/AIDS Research Center (CARC) Project Toward No Drug Abuse (TND) pilot study participants, *n* = 30.

**Lifetime drug use**	**Never**	**1–2**	**3–9**	**10–19**	**20–39**	**40+**
	**% (n)**	**% (n)**	**% (n)**	**% (n)**	**% (n)**	**% (n)**
Alcohol	26.7 (8)	36.7 (11)	3.3 (1)	16.7 (5)	3.3 (1)	13.3 (4)
*Under 18*	27.3 (3)	36.4 (4)	–	18.2 (2)	9.1 (1)	9.1 (1)
*18 or Older*	26.3 (5)	36.8 (7)	5.3 (1)	15.8 (3)	–	15.8 (3)
*Male*	42.1 (8)	31.6 (6)	–	15.8 (3)	–	10.5 (2)
*Female*	–	45.5 (5)	9.1 (1)	18.2 (2)	9.1 (1)	18.2 (2)
Cigarettes	33.3 (10)	20.0 (6)	3.3 (1)	10.0 (3)	3.3 (1)	30.0 (9)
*Under 18*	45.5 (5)	18.2 (2)	9.1 (1)	9.1 (1)	–	18.2 (2)
*18 or Older*	26.3 (5)	21.1 (4)	–	10.5 (2)	5.3 (1)	36.8 (7)
*Male*	31.6 (6)	21.1 (4)	5.3 (1)	5.3 (1)	5.3 (1)	31.6 (6)
*Female*	36.4 (4)	18.2 (2)	-	18.2 (2)	-	27.3 (3)
Cigars or cigarillos	66.7 (20)	10.0 (3)	10.0 (3)	3.3 (1)	3.3 (1)	6.7 (2)
*Under 18*	72.7 (8)	9.1 (1)	18.2 (2)	–	–	–
*18 or Older*	63.2 (12)	10.5 (2)	5.3 (1)	5.3 (1)	5.3 (1)	10.5 (2)
*Male*	68.4 (13)	5.3 (1)	5.3 (1)	5.3 (1)	5.3 (1)	10.5 (2)
*Female*	63.6 (7)	18.2 (2)	18.2 (2)	–	–	–
Vapor product	56.7 (17)	13.3 (4)	13.3 (4)	6.7 (2)	–	10.0 (3)
*Under 18*	63.6 (7)	18.2 (2)	18.2 (2)	–	–	–
*18 or Older*	52.6 (10)	10.5 (2)	10.5 (2)	10.5 (2)	–	15.8 (3)
*Male*	52.6 (10)	10.5 (2)	21.1 (4)	5.3 (1)	–	10.5 (2)
*Female*	63.6 (7)	18.2 (2)	-	9.1 (1)	–	9.1 (1)
Marijuana	16.7 (5)	3.3 (1)	10.0 (3)	6.7 (2)	10.0 (3)	53.3 (16)
*Under 18*	27.3 (3)	–	27.3 (3)	18.2 (2)	9.1 (1)	18.2 (2)
*18 or Older*	10.5 (2)	5.3 (1)	–	–	10.5 (2)	73.7 (14)
*Male*	15.8 (3)	5.3 (1)	10.5 (2)	10.5 (2)	–	57.9 (11)
*Female*	18.2 (2)	–	9.1 (1)	–	27.3 (3)	45.5 (5)
Non-prescribed prescription drugs	76.7 (23)	6.7 (2)	3.3 (1)	6.7 (2)	–	6.7 (2)
*Under 18*	81.8 (9)	9.1 (1)	–	–	–	9.1 (1)
*18 or Older*	73.7 (14)	5.3 (1)	5.3 (1)	10.5 (2)	–	5.3 (1)
*Male*	68.4 (13)	10.5 (2)	5.3 (1)	10.5 (2)	–	5.3 (1)
*Female*	90.9 (10)	–	–	–	–	9.1 (1)

Table 4 provides a summary of alcohol, tobacco, and drug use in the prior 30 days at baseline (pre-) for the full sample and for those pilot participants who completed both the pre- and post- intervention surveys. When asked about alcohol, tobacco, and drug use in the prior 30 days at baseline, 50% of the full sample reported some alcohol use, 44.3% reported some cigarette use, and 60% reported some marijuana use. The alcohol and marijuana use rates among our pilot participants were higher than both the national and Southern MSA region NIDA-MTF estimates. In the national sample, 79.5% of surveyed individuals reported not using alcohol, and 82.3% of the Southern MSA region survey participants reported no alcohol use. In the national sample, 76.3% of participants reported not using marijuana, and there were similar levels of non-use reported in the Southern MSA sample (77.5%), compared to 40.0% self-reporting non-use of marijuana in our CARC TND pilot sample.

**Table 4:** Pre and post-intervention estimates of alcohol, tobacco, marijuana, and other drug use in the last 30 days among Comprehensive Alcohol-HIV/AIDS Research Center (CARC) Project Toward No Drug Abuse (TND) pilot study participants.

**30 Day drug use**	**0 times**	**1–2**	**3–9**	**10–19**	**20–39**	**40+**
	**% (n)**	**% (n)**	**% (n)**	**% (n)**	**% (n)**	**% (n)**
**Alcohol**						
**TND**						
*Pre (n = 30)*	50.0 (15)	50.0 (15)	–	–	–	–
*Pre (n = 8)*	75.0 (6)	25.0 (2)	–	–	–	–
*Post (n = 8)*	62.5 (5)	37.5 (3)	–	–	–	–
**NIDA-MTF**						
*AA Southern MSA (n = 768)*	82.3 (632)	12.9 (99)	4.4 (34)	0.1 (1)	–	0.2 (2)
*National AA (n = 1,543)*	79.5 (1,226)	13.5 (208)	5.6 (87)	0.6 (10)	0.3 (4)	0.5 (8)
**Cigarettes**						
**TND**						
*Pre (n = 30)*	56.7 (17)	13.3 (4)	–	13.3 (4)	6.7 (2)	10.0 (3)
*Pre (n = 8)*	75.0 (6)	12.5 (1)	–	–	12.5 (1)	–
*Post (n = 8)*	87.5 (7)	12.5 (1)	–	–	–	–
**Marijuana**						
**TND**						
*Pre (n = 30)*	40.0 (12)	20.0 (6)	–	3.3 (1)	6.6 (2)	30.0 (9)
*Pre (n = 8)*	50.0 (4)	37.5 (3)	–	–	–	12.5 (1)
*Post (n = 8)*	25.0 (2)	37.5 (3)	–	12.5 (1)	12.5 (1)	12.5 (1)
**NIDA-MTF**						
*AA Southern MSA (n = 782)*	77.5 (606)	7.0 (55)	6.1 (48)	3.1 (24)	2.4 (19)	3.8 (30)
*All AA (n = 1,589)*	76.3 (1,213)	7.4 (117)	6.2 (99)	2.8 (45)	2.2 (35)	5.0 (80)

*No reported pre- or post-intervention use of cocaine, hallucinogens, stimulants, or inhalants in the past 30 days. Change in 30-day use not statistically significant between pre- and post-intervention, assessed by paired t-test*.

When looking at differences by participation, participants who attended 50% or more of the intervention sessions also completed the post-intervention survey at a frequency of 26.7%. There were few differences between individuals who completed both pre- and post-surveys. In substance use reported in the 30 days prior to the survey, there was an increase in the use of alcohol. One participant reported new alcohol use between the pre- and post-intervention survey. In addition, one less pilot participant reported using cigarettes in 30 days prior to completing the survey.

When comparing pre- and post-intervention survey estimates for intentions of future use of alcohol, tobacco, marijuana, and other drugs in the next 12 months, we observed some increases in CARC TND pilot participants reporting that they definitely would not use alcohol, cigarettes, or other drugs. However, these estimates were not statistically significant (Supplemental Table 2). Of the 30 participants, only 8 participants completed both the pre- and post-intervention survey, which would provide the ability to compare changes in participants responses. Following the intervention, 50% of the CARC TND pilot participants stated they definitely would not use alcohol in the next 12 months compared to 37.5% pre-intervention. For cigarettes, 87.5% reported they definitely would not use after the intervention, compared to 75% pre-intervention. Of those who completed the post-intervention survey, all reported they were not anticipating using any other drugs within the next 12 months after the intervention, compared to 87.5% pre-intervention.

## Discussion

Our pilot study population focused on youth and young adults in New Orleans, Louisiana, who are some of the most at-risk and underserved in the nation. Youth in Louisiana are more likely to drop out of school without a high school diploma than youth nationwide, and New Orleans youth are more likely to live in poverty than youth in Louisiana and nationwide (20). We pilot tested the feasibility of implementing the CARC TND drug education curriculum in a predominantly African American, out-of-school, urban young adult population in a social services community program. We were able to adapt a 14-session alcohol and drug use intervention schedule to cover all material in 12 weekly sessions. The program was acceptable and highly rated among the participants. However, attendance and retention were low. Results from our pilot study emphasize the need to devote additional educational resources to reinforce behavior change and personnel to increase retention of vulnerable youth in an intervention program.

Although the TND program had been tested extensively in Hispanic/Latino populations (22), the efficacy of the program in an African American majority population not in school had not been previously reported in the literature. Our analysis provides insight into the applicability of TND material for out-of-school and young adults who may have had greater occupational responsibilities and environmental stressors than a typical high school student. Our target population had a higher rate of alcohol and drug use when compared to national samples. Therefore, this was a target population that was underserved and not effectively targeted by current drug abuse prevention interventions. The TND curriculum was flexible and adaptable to a non-traditional school setting for older youth. From the literature, it is evident that a challenge remains in adapting a validated intervention for different populations with different cultural needs, while maintaining fidelity (23). We tried to maintain the original materials and session aids as much as possible. However, to improve content delivery, an optional TND video was omitted due to outdated materials based on observations by the study team and feedback from participant focus groups. In addition, some scheduling modifications were incorporated to fit with existing program schedules in collaboration with New Orleans YEP programming.

The differences of perceived applicability of the program curriculum between traditional high school TND students and the YEP community program highlighted components that need to be considered when targeting an older young adult population. A continual challenge that affected the potential effect of the CARC TND program on decreasing alcohol, tobacco, and drug use among young adults was inconsistent attendance at the intervention sessions. Participation was not coupled with mandatory classroom attendance as it would be in traditional high schools. The pilot participants, as part of the YEP program, were simultaneously enrolled in GED/HiSET courses, tended to live independently, and held employment positions in the community; these time demands are not typical of traditional high school students. Transportation issues were identified as a recurring barrier that contributed to poor attendance and retention. Although participants were offered compensation for their time participating in the pilot program and attending intervention sessions, retention in the program was still low. Unfortunately, due to low retention with our pilot sample size, we were not able to completely evaluate the effect of the program on different age groups or determine any difference in effect by sex. This is a definite area in need of further expansion and re-evaluation.

Additionally, due to program schedules and availability of YEP services, the content was delivered in weekly sessions instead of 2–3 times a week as originally designed. This adaptation may affect learning and retention of material in comparison to more structured school settings. Assessing the efficacy of weekly course scheduling for TND implementation provides an important emphasis about the adaptability of the program to less rigid settings than a high school classroom, but there needs to be reinforcement of concepts and promotion of changes to improve health-related behaviors. We have started to explore the incorporation of providing supplemental materials and reminders via mobile smartphone technologies as a potential option for additional educational resources to increase effectiveness of interventions.

Some additional insight into the lack of the intervention affecting alcohol, tobacco, and marijuana use is that participants in our TND pilot program had significantly higher baseline use of these substances than those reported nationally. The most commonly reported substances among our CARC TND Pilot participants included alcohol and tobacco, substances that are highly associated with the development of substance use disorders (24). Based on our evaluation of findings, it is recommended that interventions for older youth and those not in a traditional school setting require greater educational focus on decreasing harmful (high-risk) substance use and facilitating linkage to cessation, rather than heavy focus on preventing commencement of use. This shift in focus will reflect greater application to those who already use substances and may increase relevance of the program to these individuals. The design of an intervention program for older adolescents in our community setting may be served by an additional emphasis on safe alcohol consumption practices, as opposed to abstinence. Ultimately, the goal of TND is to prevent abuse of substances. Therefore, a focus on safe drinking practices may be more relevant for young adults of legal drinking age, which still meets the goal of CARC TND.

In summary, with high rates of use, there is a need for effective intervention programs for decreasing alcohol, tobacco, and substance use among out-of-school youth. Through our CARC TND pilot program, we were able to explore the feasibility and relevance of an existing substance abuse educational program in a new population and community setting. Although it was well-received by our urban African American young adult target population, the CARC TND intervention did not result in significant decreases in self-reported 30-day alcohol, cigarette, or illicit drug use. Upon evaluation by our team, we feel that participants with time constraints and competing priorities would benefit from additional re-enforcement of material from 1 week to the next as well as additional retention strategies.

## Data Availability Statement

The datasets generated for this study are available on request to the corresponding author.

## Ethics Statement

The studies involving human participants were reviewed and approved by LSUHSC Institutional Review Board. Written informed consent to participate in this study was provided by the participants' legal guardian/next of kin.

## Author Contributions

KT, NG, PM, and SE conceptualized the research design and AR coordinated the research study. TF, AB, ER, and AR analyzed data and wrote the manuscript with contributions from AR, KT, NG, PM, and SE. All authors contributed to the article and approved the submitted version.

## Conflict of Interest

The authors declare that the research was conducted in the absence of any commercial or financial relationships that could be construed as a potential conflict of interest.
