# Supplementary material for: Pilot Study of the Adaptation of an Alcohol, Tobacco, and Illicit Drug Use Intervention for Vulnerable Urban Young Adults
Source: Front Public Health. 2020 Jul 17;8:314. doi: 10.3389/fpubh.2020.00314 (PMC7379478; doi:10.3389/fpubh.2020.00314)
Supplement: Supplementary file 1 [file Data_Sheet_1.docx]

**Supplemental Table 1.** Baseline number of times in their lifetime substances were used among Project Towards No Drug Abuse (TND) pilot study participants, n=30.

| **Lifetime Drug Utilization** | | **Never** | **1-2** | **3-9** | **10-19** | **20-39** | **40+** |
| --- | --- | --- | --- | --- | --- | --- | --- |
|  | | **%(n)** | **%(n)** | **%(n)** | **%(n)** | **%(n)** | **%(n)** |
| **Alcohol** | 26.7 (8) | | 36.7 (11) | 3.3 (1) | 16.7 (5) | 3.3 (1) | 13.3 (4) |
| **Cigarettes** | 33.3 (10) | | 20.0 (6) | 3.3 (1) | 10.0 (3) | 3.3 (1) | 30.0 (9) |
| **Cigars or cigarillos** | 66.7 (20) | | 10.0 (3) | 10.0 (3) | 3.3 (1) | 3.3 (1) | 6.7 (2) |
| **Vapor product** | 56.7 (17) | | 13.3 (4) | 13.3 (4) | 6.7 (2) | - | 10.0 (3) |
| **Marijuana** | 16.7 (5) | | 3.3 (1) | 10.0 (3) | 6.7 (2) | 10.0 (3) | 53.3 (16) |
| **Non-prescribed Prescription drugs** | 76.7 (23) | | 6.7 (2) | 3.3 (1) | 6.7 (2) | - | 6.7 (2) |
|  |  | |  |  |  |  |  |

**Supplemental Table 2.** Pre and Post-Test estimates plans for future use of alcohol, tobacco, marijuana, and others drug use in the next 12 months among Project Towards No Drug Abuse (TND) pilot study participants.

| **Plans for use in the next 12 months** | | |  |  |  |  |
| --- | --- | --- | --- | --- | --- | --- |
|  | | **Definitely not** | **Probably Not** | **A little likely** | **Somewhat Likely** | **Very Likely** |
|  |  | *% (n)* | *% (n)* | *% (n)* | *% (n)* | *% (n)* |
| **Alcohol** |  |  |  |  |  |  |
| *Pre (n=29)* |  | 37.9 (11) | 17.2 (5) | 31.0 (9) | 13.8 (4) | - |
| *Pre with a Post (n=8)* |  | 37.5 (3) | 12.5 (1) | 37.5 (3) | 12.5 (1) | - |
| *Post (n=8)* |  | 50.0 (4) | 12.5 (1) | 37.5 (3) | - | - |
| **Cigarettes** |  |  |  |  |  |  |
| *Pre (n=29)* |  | 65.5 (19) | 10.3 (3) | 6.9 (2) | 6.9 (2) | 10.3 (3) |
| *Pre with a Post (n=8)* |  | 75.0 (6) | 25.0 (2) | - | - | - |
| *Post (n=8)* |  | 87.5 (7) | - | 12.5 (1) | - | - |
| **Marijuana** |  |  |  |  |  |  |
| *Pre (n=29)* |  | 20.7 (6) | 17.2 (5) | 13.8 (4) | 13.8 (4) | 34.5 (10) |
| *Pre with a Post (n=8)* |  | 25.0 (2) | 25.0 (2) | - | 37.5 (3) | 12.5 (1) |
| *Post (n=8)* |  | - | 25.0 (2) | 37.5 (3) | 25.0 (2) | 12.5 (1) |
| **Any other drug** |  |  |  |  |  |  |
| *Pre (n=29)* |  | 93.1 (27) | - | - | 6.9 (2) | - |
| *Pre with a Post (n=8)* |  | 87.5 (7) | - | - | 12.5 (1) | - |
| *Post (n=8)* |  | 100.0 (8) | - | - | - | - |
|  |  |  |  |  |  |  |
